# Psychotic symptoms in danon disease: A clinical case report

**DOI:** 10.1192/j.eurpsy.2021.1950

**Published:** 2021-08-13

**Authors:** C. Massaneda Tuneu, V. Soria, J. Gascón-Bayarri

**Affiliations:** 1 1. department Of Psychiatry, Bellvitge University Hospital- IDIBELL, Hospitalet del Llobregat, Barcelona, Spain; 2 Faculty Of Medicine And Health Sciences, University of Barcelona, Barcelona, Spain; 3 4. network Center For Biomedical Research On Mental Health (cibersam), Carlos III Health Institute (ISCIII), Barcelona, Spain; 4 Department Of Neurology, Bellvitge University Hospital-IDIBELL, Hospitalet del Llobregat, Barcelona, Spain

**Keywords:** psychosis, neurology, danon disease

## Abstract

**Introduction:**

Danon disease is an X-linked cardioskeletal myopathy related to a primary deficiency in lysosome-associated membrane protein-2. Danon disease manifests with the triad of hypertrophic cardiomyopathy, myopathy and intellectual disability.Psychiatric symptoms related to the disease have only been studied in a few case reports (Hatz et al, 2010 and Tanidir et al, 2015) and a case series (Yardeni et al, 2016), leaving its pathophysiological mechanisms understudied.

**Objectives:**

Provide scientific data on psychotic symptoms in patients with Danon disease.

**Methods:**

We report an unusual case of a 25-year-old-patient affected by Danon disease that showed an acute psychotic episode.

**Results:**

Mr P is a 25 year-old white male, with past medical history for Danon disease. Mr P presents hypertrophic cardiomyopathy, Wolf Parkinson White arrhythmia and carries an implantable cardioverter-defibrillator. There are previous records of mild intellectual disability and the patient had experienced anxiety symptoms as well as obsessive thoughts in the past without receiving any specific diagnosis or treatment. He was admitted to the Neurology inpatient unit to study behavioural symptoms with atypical visual and auditory hallucinations, accompanied by paranoid delusions during the last 4 days. He was examined by the liaison psychiatric team. Psychosis remitted within 72 hours after introducing risperidone 3mg per day, with good tolerability. Magnetic resonance imaging (MRI) scan was normal.

**Conclusions:**

Danon disease is caused by heterogeneity genetic mutation which means that patients can present different levels of clinical manifestations. The current case report highlights the variety of psychiatric symptoms in patients with Danon disease, and raises awareness towards its identification and treatment.

**Disclosure:**

No significant relationships.

